# Magnetic sphincter augmentation in the management of gastro-esophageal reflux disease: a systematic review and meta-analysis

**DOI:** 10.1097/JS9.0000000000001558

**Published:** 2024-05-09

**Authors:** Michael G. Fadel, Munir Tarazi, Madhav Dave, Marcus Reddy, Omar Khan, Naim Fakih-Gomez, Hutan Ashrafian, Matyas Fehervari

**Affiliations:** a Department of Surgery and Cancer, Imperial College London; b Department of Bariatric and Metabolic Surgery, Chelsea and Westminster Hospital, London; c Department of Upper Gastrointestinal Surgery, Liverpool University Hospitals, Liverpool; d Department of Bariatric Surgery; e Population Sciences Department, St George’s University of London; f Department of Gastrointestinal Surgery, Maidstone and Tunbridge Wells NHS Trust, United Kingdom

**Keywords:** efficacy, fundoplication, gastro-esophageal reflux disease, magnetic sphincter augmentation, outcomes

## Abstract

**Background:**

Magnetic sphincter augmentation (MSA) through placement of the LINX device is an alternative to fundoplication in the management of gastro-esophageal reflux disease (GERD). This systematic review and meta-analysis aimed to assess efficacy, quality of life, and safety in patients that underwent MSA, with a comparison to fundoplication.

**Methods:**

A literature search of MEDLINE, Embase, Emcare, Scopus, Web of Science, and Cochrane library databases was performed for studies that reported data on outcomes of MSA, with or without a comparison group undergoing fundoplication, for GERD from January 2000 to January 2023. Meta-analysis was performed using random-effect models and between-study heterogeneity was assessed.

**Results:**

Thirty-nine studies with 8075 patients were included: 6983 patients underwent MSA and 1092 patients had laparoscopic fundoplication procedure. Ten of these studies (seven retrospective and three prospective) directly compared MSA with fundoplication. A higher proportion of individuals successfully discontinued proton-pump inhibitors (*P*<0.001; WMD 0.83; 95% CI: 0.72–0.93; *I* ^2^=96.8%) and had higher patient satisfaction (*P*<0.001; WMD 0.85; 95% CI: 0.78–0.93; *I* ^2^=85.2%) following MSA when compared to fundoplication. Functional outcomes were better after MSA than after fundoplication including ability to belch (*P*<0.001; WMD 0.96; 95% CI: 0.93–0.98; *I* ^2^=67.8) and emesis (*P*<0.001; WMD 0.92; 95% CI: 0.89–0.95; *I* ^2^=42.8%), and bloating (*P*=0.003; WMD 0.20; 95% CI: 0.07–0.33; *I* ^2^=97.0%). MSA had higher rates of dysphagia (*P*=0.001; WMD 0.41; 95% CI: 0.17–0.65; *I* ^2^=97.3%) when compared to fundoplication. The overall erosion and removal rate following MSA was 0.24% and 3.9%, respectively, with no difference in surgical reintervention rates between MSA and fundoplication (*P*=0.446; WMD 0.001; 95% CI: −0.001–0.002; *I* ^2^=78.5%).

**Conclusions:**

MSA is a safe and effective procedure at reducing symptom burden of GERD and can potentially improve patient satisfaction and functional outcomes. However, randomized controlled trials directly comparing MSA with fundoplication are necessary to determine where MSA precisely fits in the management pathway of GERD.

## Introduction

Highlights

- Systematic review and meta-analysis assessing efficacy, quality of life, and safety in patients that underwent magnetic sphincter augmentation (MSA), with a comparison to fundoplication for gastro-esophageal reflux disease (GERD).
- Thirty-nine studies of 8075 patients were included, with 10 of these studies directly comparing MSA with fundoplication.
- MSA is a safe and effective procedure for reducing symptom burden of GERD and can potentially improve patient satisfaction and functional outcomes.
- Randomized controlled trials directly comparing.

Gastro-esophageal reflux disease (GERD) is the most common gastrointestinal condition, affecting up to 28% of the Western population^1–3^. It represents a significant burden on the healthcare system reducing patient’s health-related quality of life, whilst increasing the risk of developing Barrett’s metaplasia and therefore esophageal adenocarcinoma^4^. Initial management options include lifestyle changes and acid suppression therapy, including proton-pump inhibitors (PPIs) or H2 receptor antagonists. Despite this, nearly 40% of patients can still experience persistent symptoms^5–7^. Uncontrolled esophageal acid exposure may subsequently lead to major complications such as esophagitis, peptic stricture, aspiration pneumonia, pulmonary fibrosis, exacerbations of chronic obstructive lung disease, and esophageal adenocarcinoma^8,9^.

Laparoscopic fundoplication is the current surgical gold standard for the treatment of GERD, which can be performed either as a 360^o^ (Nissen) or a partial (Toupet or anterior). This procedure is generally safe, effective, and durable if performed in specialized centers with the correct indications and appropriate techniques^10^. However, fundoplication is often underused due to the perceived technical difficulties and potential adverse effects such as bloating, inability to belch or emesis, dysphagia, recurrent symptoms, and anatomic failure of the repair^11^. Modification of the original Nissen fundoplication has shown some reduction in adverse effects, such as dysphagia, when partial fundoplication techniques are employed^12^. However, these partial fundoplication procedures are associated with prolonged use of PPIs in ~60% of patients, and a proportion of patients (5–10%) still experience postoperative dysphagia. In addition, it has been shown that up to 3% of patients may require surgical reintervention within 90 days of fundoplication, and 4–6% of patients may require reintervention over the long-term^12–15^. Despite the high prevalence of GERD, fundoplication procedures are therefore often reserved for physically fit patients with chronic GERD or those with large hiatal hernias.

In 2007, the LINX magnetic sphincter augmentation (MSA) device was introduced as a minimally invasive alternative to fundoplication. This procedure involves laparoscopic placement of dynamic magnetic beads at the distal esophagus to augment lower esophageal tone to prevent reflux, whilst simultaneously preserving gastric anatomy. An early number of randomized controlled trials (RCTs), observational studies and review articles demonstrated that MSA is superior at controlling symptoms of GERD when compared to PPIs^16,17^. Further research has suggested that MSA can lead to an improvement in patient-reported health-related quality of life (GERD-HRQL) outcomes, with low rates of postoperative gas-bloating syndrome and inability to belch and/or emesis^2,16–20^.

Since the Coronavirus (COVID-19) pandemic, surgical practice has significantly been impacted. The pandemic has had ramifications on surgical techniques, open/minimally invasive procedures, theater workflow, patient and staff safety, education, and training. Innovative surgical advancements which enhance patient safety and are cost-effective are therefore increasingly being required. Both the National Institute for Health and Care Excellence (NICE), in the United Kingdom, and the American Gastroenterological Association (AGA), in the United States, now recommend the MSA device as an alternative option to fundoplication. These organizations advocate for offering MSA insertion in some patients with GERD due to its demonstrated efficacy and favorable safety profile^21,22^. At present, it is now understood that ~50 000 patients have undergone the MSA procedure internationally^23^.

The aim of this systematic review and meta-analysis is to provide updated evidence-based guidance by assessing the clinical outcomes of MSA and providing a comparison between MSA and fundoplication in the management of GERD. The main outcome measures include the impact of symptom burden associated with GERD, quality of life, procedural complications, and reinterventions. Operative time, length of stay, rate of device erosion, and removal from included studies are also presented.

## Methods

### Search strategy

A literature search of MEDLINE, Embase, Emcare, Scopus, Web of Science, and Cochrane library databases was performed. Specific search equations were formulated for each database using the following Medical Subject Headings (MeSH) terms: LINX device, magnetic sphincter augmentation, fundoplication, Nissen fundoplication, Toupet fundoplication, laparoscopy, gastro-esophageal reflux disease, efficacy, quality of life, and safety. We retrieved articles published in the English language between 1st January 2000 and 1st January 2023 that reported clinical outcomes of MSA, and compared MSA with fundoplication, in the treatment of GERD. The reference lists from the selected studies were reviewed to identify any additional relevant studies. The full search strategy is presented in Supplementary Table S1 (Supplemental Digital Content 4, http://links.lww.com/JS9/C512).

The work has reported in line with Preferred Reporting Items for Systematic Reviews and Meta-Analyses (PRISMA) (Supplemental Digital Content 1, http://links.lww.com/JS9/C509) (Supplemental Digital Content 2, http://links.lww.com/JS9/C510)^24^ and Assessing the methodological quality of systematic reviews (AMSTAR) (Supplemental Digital Content 3, http://links.lww.com/JS9/C511) Guidelines^25^. The study was registered in the PROSPERO database (CRD42023421048) for systematic reviews in April 2023.

### Study selection and data extraction

All studies reporting on clinical outcomes of MSA or comparing outcomes of MSA versus fundoplication in the management of GERD were eligible for inclusion. Study types of RCTs, prospective, and retrospective studies were included in this systematic review and meta-analysis. The exclusion criteria were the following: (1) studies that published data on fundoplication or PPI therapy outcomes without comparison to MSA, (2) articles published in a non-English language or in a book, (3) letters to the editor, case reports, or conference abstracts, (4) animal studies, and (5) nonavailable full-text articles.

Two authors conducted the search and identification independently against the inclusion and exclusion criteria, arriving at a final list of articles. Any disagreement was resolved by a third independent reviewer. Each included manuscript was read to determine ultimate inclusion in the final analysis. From the manuscripts, the following information was extracted and summarized: first author, year of publication, country, study design, demographics, duration of follow-up, number of patients that underwent MSA and fundoplication, BMI, presence of hiatal hernia, and GERD-HRQL baseline. The following information was extracted and presented as forest plots analyzing data from the MSA group and fundoplication group where possible: operative time (minutes), length of stay (hours), ability to belch and/or emesis, postoperative bloating, dysphagia and PPI discontinuation, endoscopic dilatation, erosions or removal of LINX device, procedural satisfaction, and postoperative GERD-HRQL scores.

### Assessment of risk of bias

The quality of all observational studies was assessed using the Newcastle–Ottawa Scale (NOS)^26^. This was calculated by examining three factors: method of patient selection, comparability of the study groups, and number of outcomes reported. The full score was nine stars, and studies that had a score of seven stars or more were considered high quality. All studies were rated independently by two authors, with any differences resolved by consensus.

### Statistical analysis

Data analysis was performed using Stata Software, Version 15.1. StataCorp LCC, TX. Pooled weighed mean differences (WMD) and standardized mean differences (SMD) were analyzed by random-effect meta-analyses where the relevant data was available. Sub-group analysis of studies that presented MSA outcomes only was therefore performed with the extracted data for the following: (i) PPI discontinuation; (ii) patient satisfaction; (iii) dysphagia; and (iv) erosions and removal of device. Sub-group analysis of comparative studies of MSA versus fundoplication was performed with the extracted data for the following: (i) PPI discontinuation; (ii) postoperative GERD-HRQL score; (iii) patient satisfaction; (iv) operative time and length of stay; (v) dysphagia and endoscopic dilatation; (vi) ability to belch and emesis; and (vii) bloating following the two procedures. The logarithm of DerSimonian-Laird (DL) with 95% CI was used as the primary summary statistic. A *P*-value of <0.05 was deemed to be statistically significant. We applied multiple imputation approaches and nonclassical approaches of ratio-of-means^27^ to manage any missing data found. Inter-study heterogeneity was also assessed using the *I* ^2^ value to measure the degree of variation not attributable to chance alone. This was graded as low (*I* ^2^<30%), moderate (*I* ^2^=30–60%), or high (*I* ^2^>60%) based on the Cochrane Handbook for Systematic Reviews of Intervention^28^. The study results were computed and represented as forest plots.

## Results

**Figure 1:**
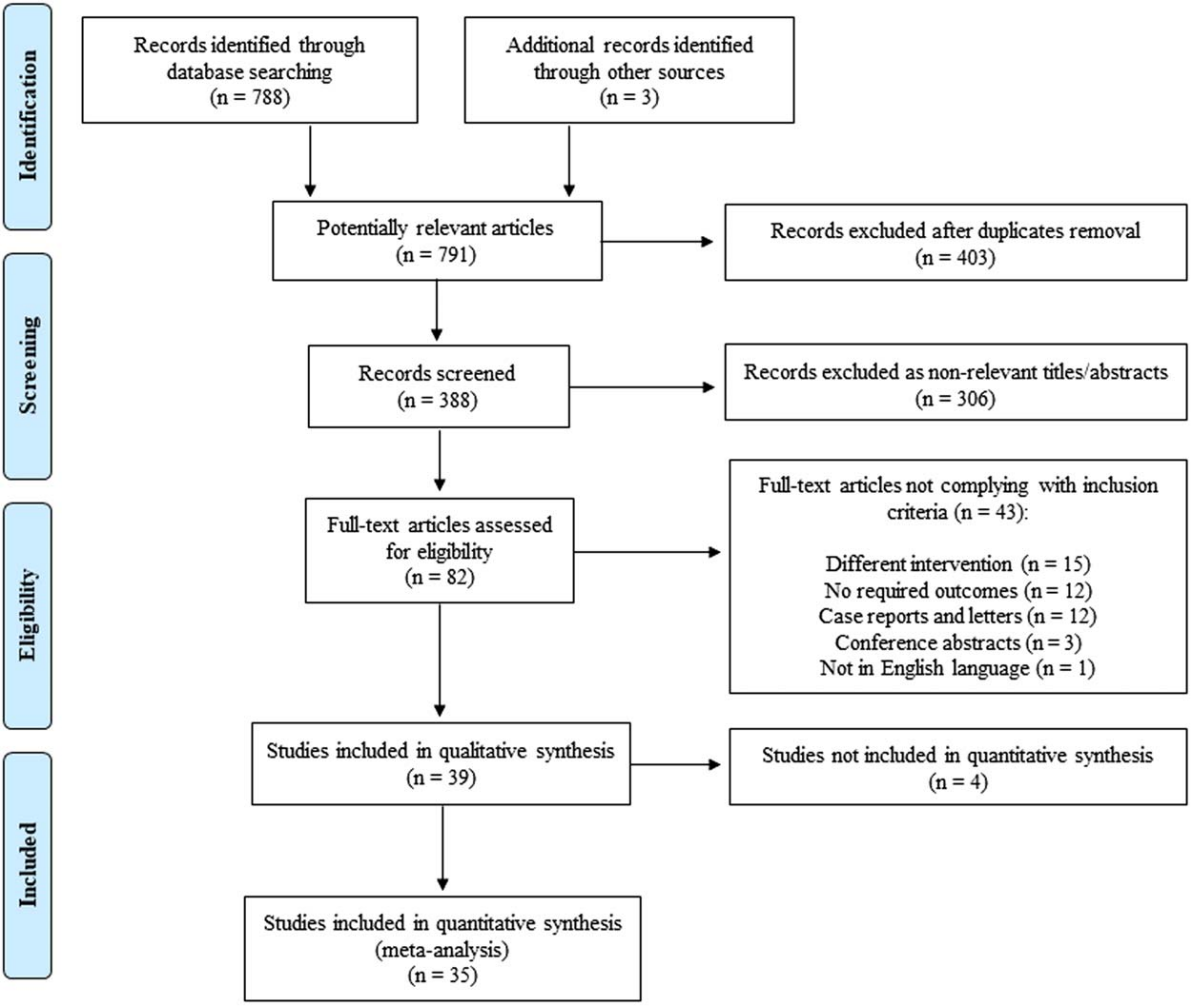
The flowchart shows the literature search and study selection process according to the PRISMA guidelines.

The initial database search and additional records identified 791 publications. A total of 709 articles were excluded after title and abstract review and removal of duplicates. Eighty-two articles were fully reviewed, and 39 and 35 studies were included in the qualitative and quantitative analysis, respectively^16,17,20,29–64^. A total of 8075 patients were included in this systematic review, comprising 6983 patients that underwent MSA and 1092 patients had a fundoplication. Ten studies were comparative studies of MSA and fundoplication, and 29 studies reported outcomes on MSA outcomes alone. The PRISMA diagram of the literature search is displayed in Figure 1.

### Baseline study and patient characteristics

The baseline study and patient characteristics, and quality assessment of the included studies are summarized in Table 1. Of the 39 studies, 25 were retrospective^20,41–64^, 12 studies^40^ were prospective and two were RCTs^16,17^. As the majority of the studies were observational studies, the NOS score was applied, and 29 studies were considered to be of low risk of bias (Supplementary Table S2, Supplemental Digital Content 4, http://links.lww.com/JS9/C512). Twenty-eight studies were from the United States, with the remaining studies from Austria, Italy, and Switzerland. The median or mean ages ranged from 42.3 years to 66.5 years and the proportion of female patients, where reported, was 49.7%. The median or mean BMI ranged from 24 to 29.1 kg/m^2^. The proportion of patients who had a hiatal hernia undergoing either MSA or fundoplication was reported in 21 studies, 2483/3542 patients (70.1%). The GERD-HRQL baseline ranged from 19.0 to 42.3 in patients that underwent MSA or fundoplication. The median or total follow-up ranged between 5 and 108 months across the studies.

**Table 1:** Study design, patient characteristics, and quality assessment of the studies included in this systematic review and meta-analysis.

Author, year	Study design (level of evidence)	Sample size (*n*)	MSA (*n*)	Fundoplication (*n*)	Median/mean age, years (range)±SD	Sex (male/female)	Median/mean initial BMI, kg/m^2^ (range)±SD	Hiatal hernia (*n*)	GERD-HRQL baseline (range)±SD	Median/total follow-up period, months	NOS
Antiporda et al^64^. 2019	Retrospective (3)	98	98	–	55±16	42/56	26.5±4.4	58	25 (18–33)	46	8*
Asti et al^40^. 2016	Prospective	238	135	103	–	–	–	–	–	44	8*
Ayazi et al^62^. 2020	Retrospective (3)	553	553	–	54.7±13.9	210/343	29±4.6	455	33.8±18.7	10.3	7*
Ayazi et al^63^. 2020	Retrospective (3)	380	380	–	55.2	137/243	29.1±4.5	335	33.7±18.9	11.5	7*
Bell et al^16^. 2019	RCT (1)	134	47	–	–	–	–	–	–	12	N/A
Bell et al^17^. 2020	RCT (1)	152	50	–	46	88/64	27.7±4.3	29	30	12	N/A
Bologheanu et al^61^. 2022	Retrospective (3)	268	268	–	50	173/95	25.5	210	19 (13–26)	23	8*
Bonavina et al^37^. 2008	Prospective (2)	38	38	–	42.8 (19–72)	23/15	24.5	23	26	12	6
Bonavina et al^38^. 2013	Prospective (2)	100	100	–	44.5	74/26	24	79	24	40	8*
Bonavina et al^39^. 2021	Prospective (2)	631	465	166	–	–	–	–	–	36	9*
Bonavina et al^60^. 2010	Retrospective (3)	44	44		42.3 (19–72)	26/18	25.7	26	25.7	24	8*
Buckley et al^36^. 2018	Prospective (2)	200	200	–	59.5	90/110	29	200	26	11	8*
Callahan et al^59^. 2023	Retrospective (3)	402	46	356	–	–	–	–	–	60	6
Czosnyka et al^58^. 2017	Retrospective (3)	102	102	–	54	53/49	28	54	27	7.6	7*
Dominguez-Profeta et al^57^. 2021	Retrospective (3)	68	68	–	51.7	27/41	25.8±4.3	45	–	26.6	8*
Dunn et al^56^. 2021	Retrospective (3)	79	79	–	66.5 (58.4–69.8)	37/42	26.32±6.93	79	21 (16.5–25.5)	35.7	8*
Ferrari et al^35^. 2021	Prospective	336	336	–	–	–	–	–	–	50.8	8*
Ferrari et al^55^. 2020	Retrospective (3)	124	124	–	44±20.8	83/41	23.9±4.5	106	21±9.5	108	8*
Ganz et al^54^. 2016	Retrospective (3)	100	100	–	53	52/48	28	–	–	60	7*
James et al^53^. 2022	Retrospective (3)	621	621	–	–	–	–	–	–	60	7*
Lipham et al^52^. 2015	Retrospective (3)	1048	1048	–	–	–	–	–	–	9	8*
Louie et al^34^. 2019	Prospective (2)	200	200	–	48.5	102/98	27.4	–	26±6.5	12	8*
Louie et al^51^. 2014	Retrospective (3)	66	34	32	–	–	–	–	–	6	6
Nikolic et al^50^. 2022	Retrospective (3)	201	201	–	51	137/64	25.5±5.5	175	20±16	22	7*
O’Neill et al^49^. 2022	Retrospective (3)	70	25	45	–	–	–	–	–	68	9*
Reynolds et al^32^. 2015	Retrospective (3)	100	50	50	–	–	–	–	–	12	7*
Reynolds et al^33^. 2014	Prospective (2)	67	67	–	53 (19–81)	47/20	–	–	–	5	6
Reynolds et al^48^. 2016	Retrospective (3)	119	52	67	–	–	–	–	–	12	8*
Riegler et al^31^. 2015	Prospective (2)	249	202	47	–	–	–	–	–	12	8*
Riva et al^47^. 2020	Retrospective (3)	45	45	–	49	32/13	–	18	19±9	12	7*
Rona et al^21^. 2017	Retrospective (3)	192	192	–	–	–	–	–	–	20	6
Saino et al^30^. 2015	Prospective (2)	44	44	–	42.8	26/18	25.7	–	25.7±6.4	60	7*
Schwameis et al^18^. 2018	Retrospective (3)	68	68	–	45	46/12	25	52	24	13	6
Schwameis et al^46^. 2021	Retrospective (3)	334	334	–	53.1	133/201	29.1±4.7	240	–	13.6	6
Sheu et al^44^. 2015	Retrospective (3)	24	12	12	–	–	–	–	–	7	8*
Smith et al^43^. 2014	Retrospective (3)	66	66	–	53.7 (18–18)	28/38	26	44	–	6	8*
Tsai et al^42^. 2020	Retrospective (3)	118	118	–	50.2	59/59	–	105	42.3	7.8	6
Warren et al^29^. 2018	Prospective	170	170	–	53 (43 – 60)	83/87	27 (24–30)	109	26 (19–31)	48	7*
Warren et al^41^. 2016	Retrospective (3)	415	201	214	–	–	–	–	–	12	8*

GERD-HRQL, Gastro-esophageal Reflux Disease Health-Related Quality of Life scale; MSA, magnetic sphincter augmentation; NOS, Newcastle–Ottawa Scale; RCT, randomized controlled trial.

– = not reported;

* = high quality studies based on Newcastle–Ottawa Scale.

### Resolution of GERD, quality of life, and patient satisfaction

**Figure 2:**
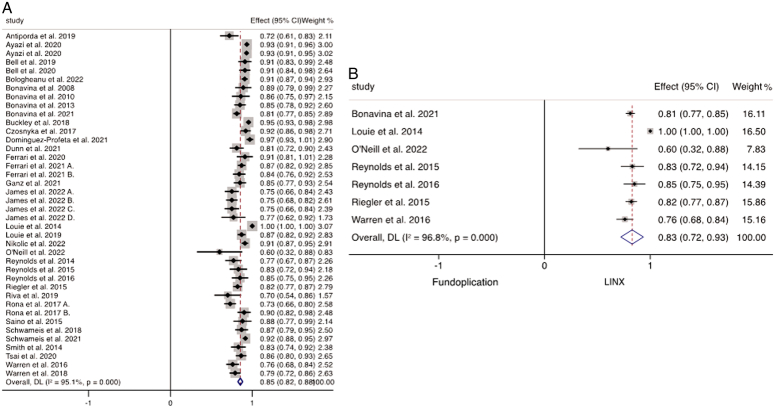
(A) Weighted mean of PPI discontinuation following MSA. (B) Meta-analysis and forest plot of studies comparing PPI discontinuation following MSA and fundoplication.

Thirty-five studies reported on the frequency of PPI discontinuation after MSA in 4850 patients. The weighted mean analysis of these studies demonstrated that 85.4% (95% CI: 82.5–88.3%) of patients successfully discontinued PPI therapy following MSA insertion (Fig. 2A). The weighted mean of DeMeester score following MSA insertion was 13.6 (95% CI: 13.4–13.9). Seven studies directly compared the rates of PPI discontinuation between the different antireflux surgeries, including 794 patients who underwent MSA and 449 patients who had fundoplication. A random-effects analysis revealed a significantly higher proportion of individuals who successfully discontinued PPIs following MSA insertion across these studies (*P*<0.001; WMD 0.83, 95% CI: 0.72–0.93, *I* ^2^=96.8%) (Fig. 2B).

**Figure 3:**
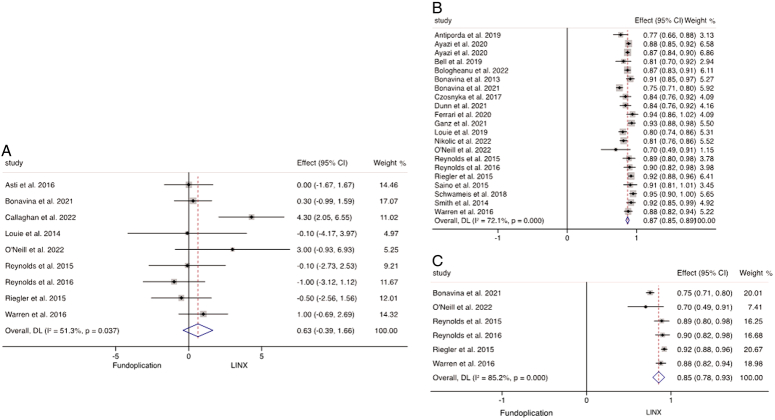
(A) Meta-analysis and forest plot of studies comparing GERD-HRQL following MSA and fundoplication. (B) Weighted mean of patient satisfaction following MSA. (C) Meta-analysis and forest plot of studies comparing patient satisfaction following MSA and fundoplication.

The weighted mean of the postoperative GERD-HRQL score of 4804 patients undergoing MSA was 4.57 (95% CI: 4.5–4.6). When comparing the postoperative GERD-HRQL scores after MSA and fundoplication, there were no significant differences found between the scores following the two procedures (*P*=0.224; WMD 0.63; 95% CI: −0.39–1.66; *I* ^2^=51.3%) (Fig. 3A). Patient satisfaction following MSA was assessed in 21 studies reporting outcomes for 2895 patients which demonstrated that 87.0% (95% CI: 85.0–89.5%) were satisfied with the outcome of the operation within the first 2 years (Fig. 3B). Six studies directly compared MSA and fundoplication groups, which revealed that patient satisfaction was significantly higher following MSA (*P*<0.001; WMD 0.85; 95% CI: 0.78–0.93; *I* ^2^= 85.2%) (Fig. 3C).

### Perioperative outcomes

**Figure 4:**
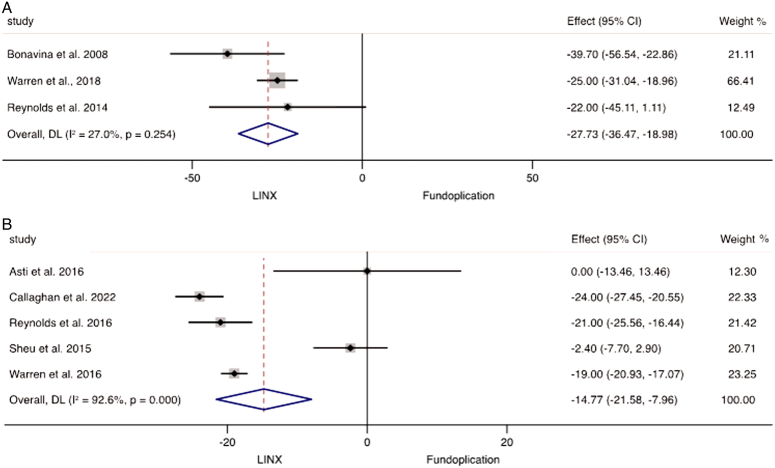
Meta-analysis and forest plot of studies comparing weighted mean difference of (A) operative time and (B) length of stay following MSA and fundoplication.

The operative times and length of stay of MSA versus fundoplication were sporadically reported across the 39 studies. Based on three studies of 719 patients, MSA had a statistically significant shorter operative time (*P*<0.001; WMD −27.7; 95% CI: −36.5 to −19.0; *I* ^2^=27.0%) (Fig. 4A) and length of stay based on 1186 patients in five studies (*P*<0.001; WMD −14.8; 95% CI: −21.6 to −8.0; *I* ^2^=92.6%) (Fig. 4B). This equates to the MSA procedure being on average 27.7 min shorter than fundoplication with a reduced length of stay by 14.8 h across these studies. Postoperative mortality was investigated in 34 studies, including 5866 individuals undergoing MSA insertion and 767 patients having fundoplication, and there was no postoperative mortality encountered in any of the studies.

### Dysphagia

**Figure 5:**
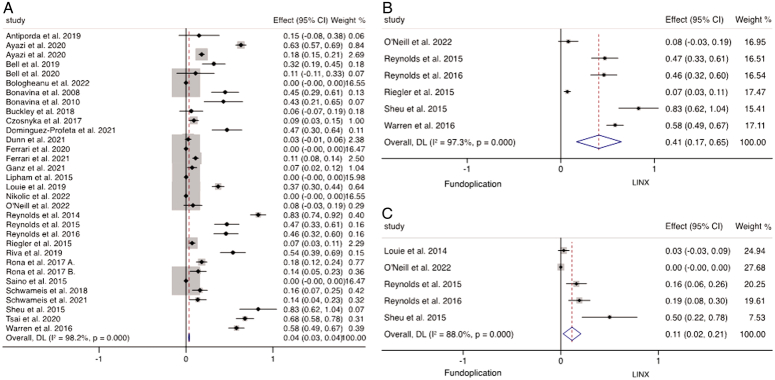
Meta-analysis and forest plot of studies of weighted mean of (A) dysphagia following MSA. Meta-analysis and forest plot of studies comparing weighted mean difference of (B) dysphagia and (C) endoscopic dilatation following MSA and fundoplication.

Thirty-two studies including 4324 patients reported on the incidence of postoperative dysphagia following MSA. The weighted mean of these studies suggested that 4.0% (95% CI: 3.0–4.0%) reported dysphagia following MSA insertion (Fig. 5A). Twenty-nine studies investigating 5448 MSA insertions reported the frequency of postoperative endoscopic dilatation. The weighted means of these studies suggested that 11.0% (95% CI: 8.9–13.5%) required endoscopic dilatation to relieve symptoms of dysphagia. There were six studies, including 772 patients (MSA 448; fundoplication 324), comparing the outcomes of MSA directly to fundoplication. This direct comparison demonstrated higher rates of dysphagia following MSA when compared to fundoplication (*P*=0.001; WMD 0.41; 95% CI: 0.17–0.65; *I* ^2^=97.3%) (Fig. 5B). There were five studies of 361 patients (MSA 166; fundoplication 195) comparing the frequency of postoperative endoscopic dilatation between MSA and fundoplication. Random-effect analysis of these studies suggested significantly more dilatation required in the MSA group (*P*=0.013; WMD 0.11; 95% CI: 0.02–0.21; *I* ^2^=88.0%) (Fig. 5C).

### Functional outcomes

**Figure 6:**
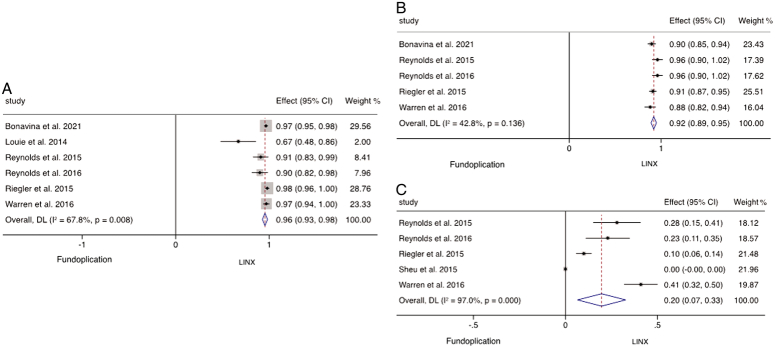
Meta-analysis and forest plot of studies assessing (A) ability to belch, (B) ability to emesis, (C) bloating following MSA and fundoplication treatment.

Fifteen studies, involving a total of 1817 patients, examined the ability to belch following MSA. The pooled analysis of these studies revealed that 99.7% (95% CI: 99.4–100%) of patients retained the ability to belch after undergoing MSA. Comparing the ability to belch following MSA and fundoplication were reported by six studies comprising of 1580 patients (MSA 841; fundoplication 422). Random-effect analysis revealed that significantly more patients maintained the ability to belch following MSA (*P*<0.001; WMD 0.96; 95% CI: 0.93–0.98; *I* ^2^=67.8%) (Fig. 6A). There were 13 studies of 1504 patients investigating the ability to emesis, with 80.1% (95% CI: 72.6–87.5%) of patients maintaining the ability to emesis with MSA. There was a difference found in the ability to emesis between the two groups in five studies (*P*<0.001; WMD 0.92; 95% CI: 0.89–0.95; *I* ^2^=42.8%) (Fig. 6B). In the 13 studies of 696 patients undergoing MSA, bloating was reported in 2.9% (95% CI: 1.8–4.0%). There was significantly less postoperative bloating following MSA reported when compared to fundoplication in five studies (*P*=0.003, WMD 0.20; 95% CI: 0.07–0.33; *I* ^2^=97.0%) (Fig. 6C).

### Surgical reintervention, removal, and erosion

**Figure 7:**
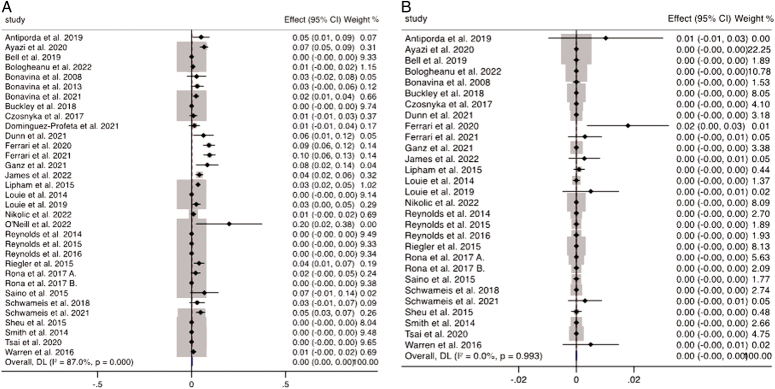
Meta-analysis and forest plot of studies assessing (A) erosions and (B) removal of MSA device.

There were eight studies reporting and directly comparing the need for surgical reintervention comprising of 1674 patients. Surgical reintervention was no different between the MSA and fundoplication group (*P*=0.446; WMD 0.001; 95% CI: −0.001 to −0.002; *I* ^2^=78.5%). Twenty-nine studies reported on erosions related to the MSA device (Fig. 7A), however, only six of these had included a control group of patients undergoing fundoplication. Overall, there was a total of 13 erosions from 5328 cases (0.24%). The rate of MSA removal was reported in 33 studies (Fig. 7B), however, only seven of these studies similarly included a control group. The device was removed in 232 out of 5838 cases (3.9%), with the indications of persistent dysphagia, malfunction, erosion, and the need for subsequent MRI.

## Discussion

To our knowledge, this is the largest systematic review and meta-analysis assessing the efficacy and safety of MSA, along with a comparison to fundoplication in the management of GERD. The pooled analyses of available data revealed several positive findings associated with MSA surgery. These include higher rates of PPI discontinuation, improved patient satisfaction, as well as functional outcomes. These findings are of even higher significance given the current postpandemic climate and the need for operative interventions that are minimally invasive with shorter surgical times that result in a reduced length of stay. However, it is important to note that at this stage there is a lack of robust quality comparative data on adverse events and long-term surgical reintervention, particularly explantation of MSA device following these procedures.

Previous studies demonstrated that fundoplication improves quality of life for patients undergoing fundoplication compared to medical management only^10^. Our meta-analysis highlights that MSA has the potential to further enhance patient outcomes and well-being. Regarding postoperative dysphagia, the weighted mean of 32 studies demonstrated a dysphagia rate of 3.6% after MSA. On direct comparison of up to six studies, MSA had higher rates of dysphagia and endoscopic dilatation compared to fundoplication. However, it must be noted that the majority of these six studies are dated prior to 2016, with a limited number of patients in the fundoplication group and events (particularly in the endoscopic dilatation group) and a high level of heterogeneity between the studies. The majority of the included studies also directly compared MSA to Nissen fundoplication, which is known to have a higher dysphagia rate than partial fundoplication^12^. Further studies are therefore required to directly compare MSA with partial fundoplication.

Functional outcomes (ability to belch/emesis and postoperative bloating) following MSA was found to be improved compared to fundoplication, which is consistent with other several studies^29,30,32,44,48,50,54,57,60,61,64^. This confirms the efficacy of MSA in preserving the physiological function of the upper gastrointestinal tract postoperatively. By maintaining the natural belching and emesis ability, MSA offers patients a more physiologically normal digestive experience as it allows for the release of excess gas and alleviation of potential discomfort. The improvement of these functional outcomes likely contributes to a high patient satisfaction and quality of life observed after MSA.

A higher number of adverse effects have been associated with fundoplication compared to medical management in previous studies^65,66^. The introduction of a new antireflux procedure with fewer adverse events has the potential to further enhance the treatment of GERD for patients. Surgical antireflux procedures can result in a disturbance of the anatomical configuration known as the ‘antireflux’ valve, which consists of the precise configuration of the lower esophageal sphincter (LES), the angle of His, the diaphragm, and the associated gastric and esophageal-phrenic ligaments. Following the dissection of the angle of His and the possible impairment of the sling fibers, the LES may experience a weakening effect. Furthermore, the compliance of the residual stomach can be diminished due to alterations in the gastric fundus caused by fundoplication^67–69^. A potential advantage of MSA device is that it focuses on reconstructing the function of the LES without altering gastric anatomy. As a result, it may be a suitable procedure to prevent GERD in patients with or without hiatus hernia. Additionally, there is limited evidence on the issue of long-standing GERD and its associated Barrett’s esophagus, particularly in patients with active inflammation^55^.

The frequency of device removal after MSA in this meta-analysis was similar to the previously reported frequency of surgical reintervention after fundoplication (3.9 vs. 4%)^14^. Furthermore, direct comparison of the two techniques demonstrated no difference in the frequency of surgical reintervention in this meta-analysis. The reported rate of MSA erosion was low and consistent with previous studies (0.24 vs. 0.3%), as well as the removal rate (3.9 vs. 3.3%)^70^. Migration of MSA device has been seldom reported in individual cases, with longitudinal studies with up to 5 years follow-up not encountering this adverse event^19^. A further complication highlighted in case reports specific to MSA insertion is entrapment of vagal nerve either by the device itself or scarring around the device leading to gastroparesis or syncope^71,72^. Previously, it should be noted that there were limited studies directly comparing MSA to fundoplication. The single-arm studies reported similar rates of device explantation following MSA as the reported rates of surgical reintervention following fundoplication (3.9 vs. 4–6%)^13,15^. However, it is important to note that MSA is still evolving and has the potential for further improvement in the future, including the management of dietary advice following MSA.

In its early stages, the LINX device was primarily indicated for smaller hiatus hernias. The initial proof-of-concept study published focused on participants with small hiatus hernias and without Barrett’s esophagus^73^. Although subsequent revisions have removed this contraindication, a sense of caution remains when considering the use of MSA in cases of large hiatus hernias. However, retrospective data demonstrated that short term outcomes of MSA implantation were unaffected by hiatal hernia presence and size, though larger hernias have increased recurrence. Postoperative interventions and device removal rates showed no significant differences among patients with absent, small, and large hernias, implying that the conventional belief in favor of minimal dissection for MSA implantation might need to be reconsidered^74^. Limited data is available on the role of LINX in revisional cases, however, it may offer an alternative hence comparative studies are essential to evaluate outcomes, complications, and patient satisfaction.

In addition, another important aspect of MSA is its incompatibility with MRI. The presence of the MSA device’s magnetic beads may potentially interfere with MRI, leading to complications, ineffective reflux control, or malfunction of the device. Studies have presented mixed findings, with some suggesting that MRI can be safely performed without device removal, while others recommend removal to mitigate risks^75,76^. The decision to remove the device should consider factors such as the strength of the MRI machine (up to 1.5 Tesla), the patient’s condition, the urgency of the imaging, and the risks associated with device removal. Clear guidelines are necessary to assist clinicians in making informed decisions and ensuring patient safety during MRI with the MSA device. After MSA implantation postoperative follow-up measures may include clinical check-ups and assessments. Additionally, postoperative evaluations of any complaint should initially be investigated with endoscopy to assess the status of the MSA device and to evaluate the presence or recurrence of reflux symptoms^77^.

In terms of dietary considerations, many authors advocate free diet from the first day following implantation, however, meal portion sizes may need to be adjusted after MSA implantation. This is because larger meal sizes may carry a higher likelihood of esophageal pooling in some patients who have undergone this procedure. Therefore, some patients may need to be mindful of their meal sizes and consume smaller portions to minimize this risk^1,34,78^.

In this systematic review and meta-analysis, we were unable to assess the cost-effectiveness of the LINX device as the included studies did not report sufficient data on the costs of treatment. However, an institutional assessment revealed the estimated current cost of the LINX device is ~£2500 to the National Health Service^23^. However, the overall cost of the procedure is expected to be similar to the fundoplication, as a result of the savings in theater time, equipment costs, and length of stay. The LINX procedure is on average shorter, does not require energy devices, such as the ultrasonic harmonic device, and has a reduced length of stay. Nevertheless, a detailed cost comparison of the LINX procedure and fundoplication is currently somewhat difficult as the costs are dependent on several factors, including an institution’s professional fee for the device, insurance providers, PPI costs, and the long-term costs associated with the device and the risk of explantation.

### Future prospective

The results of this meta-analysis demonstrate that MSA exhibits a comparable safety profile to fundoplication while effectively controlling symptoms associated with GERD. Hence, MSA may become a significant procedure in modern surgical management of GERD. Although erosion of the MSA device is uncommon, addressing erosion in patients can be challenging^79^. However, the studies included in this analysis did not extensively discuss the outcomes of erosion, necessitating further investigation in subsequent studies. The rate of device removal after MSA is similar to the rate of revision after fundoplication. Nevertheless, since the gastric fundus is preserved during MSA, fundoplication can still be considered as an alternative revisional procedure in the event of device failure. Similarly, MSA may emerge as an option for revisional procedures following fundoplication, which requires further exploration.

Further research is currently underway to investigate the efficacy and safety of the LINX procedure compared to fundoplication through an international multicentre double-blind phase III RCT, known as the GOLF trial^80^. A total of 460 patients are being randomised 1:1 to receive either the LINX procedure or fundoplication with a follow-up period of 24 months. The study is expected to be completed by 2028. In terms of economic benefits, MSA has been suggested to potentially have an overall cost benefit over fundoplication, which is another area that will need to be explored beyond privately funded healthcare settings^65^.

### Strengths and limitations

The strength of this systematic review and meta-analysis is that it included a large sample size of 6983 patients that underwent MSA, and the risk of bias of the included studies was found to be generally low or moderate. However, this study was subject to important limitations which must be addressed. Between-study heterogeneity was evident particularly when analyzing length of stay, bloating, dysphagia, and PPI discontinuation. In general, the majority of the studies were retrospective and presented data on MSA outcomes only, rather than providing a direct comparison between MSA and fundoplication. There were only two RCTs included, and not all the studies assessed every aspect reviewed by this meta-analysis. There was a limited number of patients included in the fundoplication group in comparison to the MSA group and the associated costs of MSA could not be evaluated amongst the studies. Insertion of the MSA device is a relatively novel procedure, which some surgeons may be technically invested in which will ultimately lead to publication of optimal outcomes and underreporting of complications. Furthermore, there was also significant variation found in the length of follow-up and follow-up protocols between the included studies.

## Conclusions

The findings of this systematic review and meta-analysis demonstrated that MSA surgery was associated with high rates of PPI discontinuation and an improvement in patient satisfaction. When compared to fundoplication, MSA had a reduced operative time and length of stay, higher rates of PPI discontinuation, and improved functional outcomes such as ability to belch/emesis and less postoperative bloating. However, there were only a few high-quality studies found that directly compared MSA with fundoplication including rates of postoperative dysphagia. RCTs are therefore required to directly compare MSA with fundoplication and to assess the long-term surgical reintervention and adverse outcomes following MSA procedures.

## Ethical approval

This study was conducted in accordance with the guidelines of our institutional research ethics committee. For this type of study (systematic review and meta-analysis), formal consent is not required.

## Sources of funding

None.

## Author contribution

M.G.F.: conceptualisation, data collection and analysis, methodology, and manuscript writing; M.T.: conceptualisation, data collection, and analysis; M.D.: conceptualisation, data collection, and analysis; M.R.: manuscript review and editing; O.K.: manuscript review and editing; N.F.G.: manuscript review, editing, and supervision; H.A.: manuscript review, editing, and supervision; M.F.: conceptualisation, data collection and analysis, methodology, manuscript review and editing, and supervision.

## Conflicts of interest disclosure

The authors declare no competing interests.

## Research registration unique identifying number (UIN)

1. Name of the registry: PROSPERO.
2. Unique identifying number or registration ID: CRD42023421048.
3. Hyperlink to your specific registration (must be publicly accessible and will be checked): https://www.crd.york.ac.uk/prospero/display_record.php?RecordID=421048

## Guarantor

Michael G. Fadel, Department of Surgery and Cancer, Imperial College London, UK. E-mail: m.fadel@imperial.ac.uk

## Data availability statement

The datasets generated and analysed during the current study are available from the corresponding author upon request.

## Provenance and peer review

Not commissioned, externally peer-reviewed.

## Supplementary Material

SUPPLEMENTARY MATERIAL
