# Supplementary figures and images for: Magnetic sphincter augmentation in the management of gastro-esophageal reflux disease: a systematic review and meta-analysis
Source: Int J Surg. 2024 May 9;110(10):6355–66. doi: 10.1097/JS9.0000000000001558 (PMC11487049; doi:10.1097/JS9.0000000000001558)

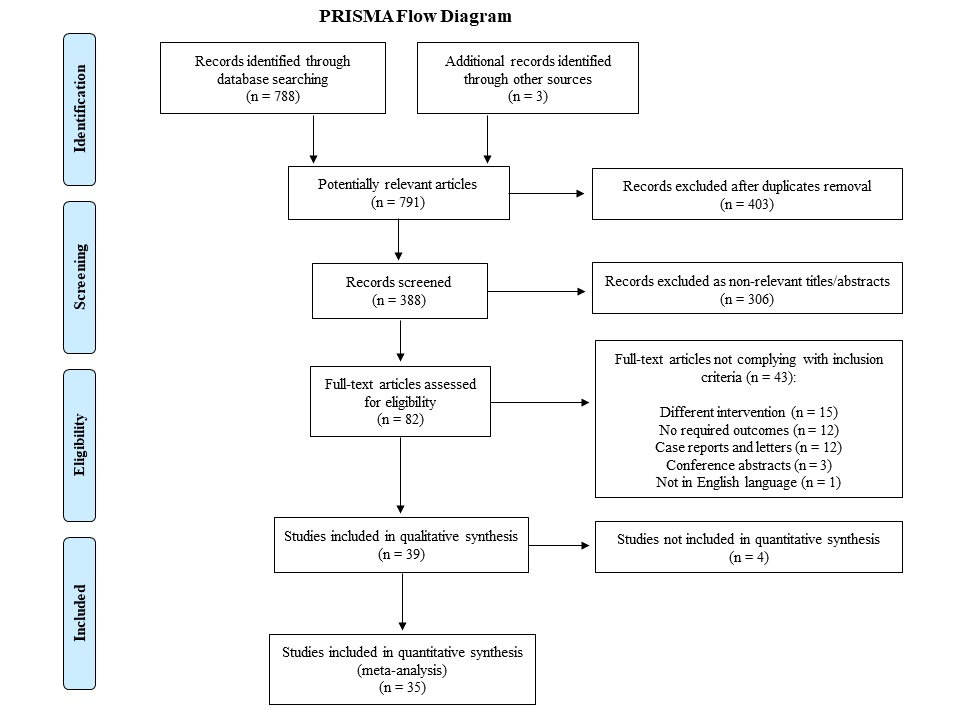

Supplement: SUPPLEMENTARY MATERIAL [file js9-110-6355-s002.jpg]
