# Supplementary material for: Magnetic sphincter augmentation in the management of gastro-esophageal reflux disease: a systematic review and meta-analysis
Source: Int J Surg. 2024 May 9;110(10):6355–66. doi: 10.1097/JS9.0000000000001558 (PMC11487049; doi:10.1097/JS9.0000000000001558)
Supplement: SUPPLEMENTARY MATERIAL [file js9-110-6355-s004.docx]

**Supplementary material**

| **Source** | **Criteria** |
| --- | --- |
| **MEDLINE**  **(Ovid)** | 1 (fundoplication or nissen fundoplication or laparoscopic nissen's fundoplication or LNF) 7740  2 Fundoplication/ 4977  3 1 or 2 7740  4 (magnetic augmentation or magnetic sphincter augmentation or MSA) 7037  5 (LINX magnetic sphincter augmentation or LINX reflux management system) 17  6 LINX 138  7 Esophageal Sphincter, Lower/su 636  8 Esophagoplasty/is, mt 1341  9 4 or 5 or 6 or 7 or 8 8983  10 (gastro-oesophageal reflux disease or gastroesophageal reflux disease or GERD or GORD or gastric reflux disease or acid reflux disease) 17813  11 Gastroesophageal Reflux/ 27718  12 10 or 11 33722  13 exp animals/ not humans.sh. 5049671  14 3 and 9 and 12 223  15 14 not 13 219  16 limit 15 to (english language and humans and yr="2000 -Current") 171  17 16 and "Case Reports".sa_pubt. 11  18 16 and "Research Support, Non-U.S. Gov't".sa_pubt. 13  19 16 and "Research Support, N.I.H., Extramural".sa_pubt. 5  20 16 and "Video-Audio Media".sa_pubt. 6  21 16 and "Comment".sa_pubt. 3  22 16 and "Letter".sa_pubt. 1  23 17 or 18 or 19 or 20 or 21 or 22 34  24 15 not 23 185  25 16 not 23 137 |
| **Embase** | 1 (fundoplication or nissen fundoplication or laparoscopic nissen's fundoplication or LNF)13850  2 stomach fundoplication/ 11426  3 1 or 2 13850  4 (magnetic augmentation or magnetic sphincter augmentation or MSA) 10887  5 (LINX magnetic sphincter augmentation or LINX reflux management system) 70  6 LINX 321  7 lower esophagus sphincter/su [Surgery] 262  8 esophagus reconstruction/ 3204  9 4 or 5 or 6 or 7 or 8 14510  10 (gastro-oesophageal reflux disease or gastroesophageal reflux disease or GERD or GORD or gastric reflux disease or acid reflux disease) 32756  11 gastroesophageal reflux/ 63410  12 10 or 11 69882  13 3 and 9 and 12 328  14 (exp animal/ or nonhuman/) not exp human/ 6896885  15 13 not 14 320  16 limit 15 to (human and english language and yr="2000 -Current") 272  17 16 and "Conference Abstract".sa_pubt. 78  18 16 and "Note".sa_pubt. 10  19 16 and "Letter".sa_pubt. 5  20 16 and "Editorial".sa_pubt. 6  21 17 or 18 or 19 or 20 99  22 16 not 21 173 |
| **Emcare** | 1 (fundoplication or nissen fundoplication or laparoscopic nissen's fundoplication or LNF) 3081  2 stomach fundoplication/ 2585  3 1 or 2 3081  4 (magnetic augmentation or magnetic sphincter augmentation or MSA) 2136  5 (LINX magnetic sphincter augmentation or LINX reflux management system) 18  6 LINX 77  7 lower esophagus sphincter/su [Surgery] 0  8 esophagus reconstruction/ 298  9 4 or 5 or 6 or 7 or 8 2469  10 (gastro-oesophageal reflux disease or gastroesophageal reflux disease or GERD or GORD or gastric reflux disease or acid reflux disease) 5557  11 gastroesophageal reflux/ 13336  12 10 or 11 14624  13 3 and 9 and 12 78  14 (exp animal/ or nonhuman/) not exp human/ 507737  15 13 not 14 77  16 limit 15 to (human and english language and yr="2000 -Current") 58  17 16 and "Conference Abstract".sa_pubt. 0  18 16 and "Note".sa_pubt. 2  19 16 and "Letter".sa_pubt. 0  20 16 and "Editorial".sa_pubt. 1  21 17 or 18 or 19 or 20 3  22 16 not 21 55 |
| **Scopus** | ( ALL ( fundoplication OR nissen AND fundoplication OR laparoscopic AND nissen's AND fundoplication OR lnf ) AND ALL ( magnetic AND augmentation OR magnetic AND sphincter AND augmentation OR msa OR linx AND magnetic AND sphincter AND augmentation OR linx AND reflux AND management AND system OR linx ) AND ALL ( gastro-oesophageal AND reflux AND disease OR gastroesophageal AND reflux AND disease OR gerd OR gord OR gastric AND reflux AND disease OR acid AND reflux AND disease ) ) AND ( LIMIT-TO ( DOCTYPE , "re" ) OR LIMIT-TO ( DOCTYPE , "ar" ) ) AND ( LIMIT-TO ( LANGUAGE , "English" ) ) |
| **Web of Science** | fundoplication or nissen fundoplication or laparoscopic nissen's fundoplication or LNF (All Fields) and magnetic augmentation or magnetic sphincter augmentation or MSA or LINX magnetic sphincter augmentation or LINX reflux management system or LINX (All Fields) and gastro-oesophageal reflux disease or gastroesophageal reflux disease or GERD or GORD or gastric reflux disease or acid reflux disease (All Fields) and Article or Review Article or Early Access (Document Types) and English (Languages) |
| **Cochrane library** | #1 fundoplication or nissen fundoplication or laparoscopic nissen's fundoplication or LNF 795  #2 MeSH descriptor: [Fundoplication] this term only 278  #3 #1 OR #2 795  #4 magnetic augmentation or magnetic sphincter augmentation or MSA 951  #5 LINX magnetic sphincter augmentation or LINX reflux management system 8  #6 LINX 19  #7 MeSH descriptor: [Esophageal Sphincter, Lower] explode all trees and with qualifier(s): [surgery - SU] 15  #8 MeSH descriptor: [Esophagoplasty] explode all trees and with qualifier(s): [instrumentation - IS, methods - MT] 7  #9 #4 OR #5 OR #6 OR #7 OR #8 983  #10 gastro-oesophageal reflux disease or gastroesophageal reflux disease or GERD or GORD or gastric reflux disease or acid reflux disease 4315  #11 MeSH descriptor: [Gastroesophageal Reflux] explode all trees 2057  #12 #10 OR #11 5001  #13 #3 AND #9 AND #12 12 |

**Table S1.** Search strategy for the systematic review and meta-analysis to identify studies reporting gastro-esophageal reflux disease outcomes following LINX magnetic sphincter augmentation versus fundoplication across six databases (MEDLINE, Embase, Emcare, Scopus, Web of Science and Cochrane library), 1^st^ January 2000 – 1^st^ January 2023.

| **Author, year** | **Selection** | | | | **Comparability** | | **Outcome** | | | **Total score** |
| --- | --- | --- | --- | --- | --- | --- | --- | --- | --- | --- |
|  | **Representativeness of the exposed cohort** | **Selection of the non- exposed cohort** | **Ascertainment of exposure** | **Outcome not present at start** | **Comparability of cohorts on the basis of the design or analysis** | **Study controls for additional factor** | **Assessment of outcome** | **Was follow-up long enough** | **Adequacy of follow up** |  |
| Antiporda et al. [63] 2019 | ✩ | ✩ | ✩ | ✩ | ✩ |  | ✩ | ✩ | ✩ | 8 |
| Antiporda et al. [64] 2019 | ✩ | ✩ | ✩ | ✩ | ✩ | ✩ |  | ✩ | ✩ | 8 |
| Asti et al. [40] 2016 | ✩ | ✩ | ✩ | ✩ | ✩ |  | ✩ | ✩ |  | 7 |
| Ayazi et al. [62] 2020 | ✩ | ✩ | ✩ | ✩ | ✩ |  | ✩ | ✩ |  | 7 |
| Ayazi et al. [63] 2020 |  |  |  |  |  |  |  |  |  | N/A* |
| Bell et al. [16] 2019 |  |  |  |  |  |  |  |  |  | N/A* |
| Bell et al. [17] 2020 | ✩ | ✩ | ✩ | ✩ | ✩ |  | ✩ | ✩ | ✩ | 8 |
| Bologheanu et al. [61] 2022 | ✩ |  | ✩ | ✩ |  |  | ✩ | ✩ | ✩ | 6 |
| Bonavina et al. [37] 2008 | ✩ | ✩ | ✩ | ✩ | ✩ |  | ✩ | ✩ | ✩ | 8 |
| Bonavina et al. [38] 2013 | ✩ | ✩ | ✩ | ✩ | ✩ |  | ✩ | ✩ | ✩ | 8 |
| Bonavina et al. [39] 2021 | ✩ | ✩ | ✩ | ✩ | ✩ | ✩ | ✩ | ✩ | ✩ | 9 |
| Bonavina et al. [60] 2010 | ✩ | ✩ | ✩ | ✩ | ✩ |  | ✩ | ✩ | ✩ | 8 |
| Buckley et al. [36] 2018 | ✩ |  | ✩ | ✩ |  |  | ✩ | ✩ | ✩ | 6 |
| Callahan et al. [59] 2023 | ✩ | ✩ | ✩ | ✩ | ✩ |  | ✩ |  | ✩ | 7 |
| Czosnyka et al. [58] 2017 | ✩ | ✩ | ✩ | ✩ | ✩ |  | ✩ | ✩ | ✩ | 8 |
| Dominguez-Profeta et al. [57] 2021 | ✩ | ✩ | ✩ | ✩ | ✩ |  | ✩ | ✩ | ✩ | 8 |
| Dunn et al. [56] 2021 | ✩ | ✩ | ✩ | ✩ | ✩ |  | ✩ | ✩ | ✩ | 8 |
| Ferrari et al. [35] 2021 | ✩ | ✩ | ✩ | ✩ | ✩ |  | ✩ | ✩ | ✩ | 8 |
| Ferrari et al. [55] 2020 | ✩ | ✩ | ✩ | ✩ | ✩ |  |  | ✩ | ✩ | 7 |
| Ganz et al. [54] 2016 | ✩ | ✩ | ✩ | ✩ | ✩ |  |  | ✩ | ✩ | 7 |
| James et al. [53] 2022 | ✩ |  | ✩ | ✩ | ✩ | ✩ | ✩ | ✩ | ✩ | 8 |
| Lipham et al. [52] 2015 | ✩ | ✩ | ✩ | ✩ | ✩ |  | ✩ | ✩ | ✩ | 6 |
| Louie et al. [34] 2019 | ✩ | ✩ | ✩ | ✩ | ✩ |  | ✩ | ✩ | ✩ | 8 |
| Louie et al. [51] 2014 | ✩ | ✩ | ✩ | ✩ | ✩ |  | ✩ |  | ✩ | 7 |
| Nikolic et al. [50] 2022 | ✩ | ✩ | ✩ | ✩ | ✩ | ✩ | ✩ | ✩ | ✩ | 9 |
| O’Neill et al. [49] 2022 | ✩ |  | ✩ | ✩ |  |  | ✩ | ✩ | ✩ | 6 |
| Reynolds et al. [32] 2015 | ✩ | ✩ | ✩ | ✩ | ✩ |  | ✩ |  | ✩ | 7 |
| Reynolds et al. [33] 2014 | ✩ | ✩ | ✩ | ✩ | ✩ |  | ✩ | ✩ | ✩ | 8 |
| Reynolds et al. [48] 2016 | ✩ | ✩ | ✩ | ✩ | ✩ |  | ✩ | ✩ | ✩ | 8 |
| Riegler et al. [31] 2015 | ✩ | ✩ | ✩ | ✩ | ✩ |  | ✩ |  | ✩ | 7 |
| Riva et al. [47] 2020 | ✩ | ✩ | ✩ | ✩ | ✩ |  | ✩ | ✩ | ✩ | 6 |
| Rona et al. [21] 2017 | ✩ | ✩ | ✩ | ✩ | ✩ |  |  | ✩ | ✩ | 7 |
| Saino et al. [30] 2015 | ✩ | ✩ | ✩ | ✩ | ✩ |  | ✩ | ✩ | ✩ | 6 |
| Schwameis et al. [18] 2018 | ✩ | ✩ | ✩ | ✩ | ✩ |  | ✩ |  |  | 6 |
| Schwameis et al. [46] 2021 | ✩ | ✩ | ✩ | ✩ | ✩ |  | ✩ | ✩ | ✩ | 8 |
| Sheu et al. [44] 2015 | ✩ |  | ✩ | ✩ |  |  | ✩ | ✩ | ✩ | 8 |
| Smith et al. [43] 2014 | ✩ | ✩ | ✩ | ✩ | ✩ |  |  | ✩ |  | 6 |
| Tsai et al. [42] 2020 | ✩ | ✩ | ✩ | ✩ | ✩ |  | ✩ | ✩ | ✩ | 8 |
| Warren et al. [29] 2018 | ✩ | ✩ | ✩ | ✩ | ✩ |  |  | ✩ | ✩ | 7 |
| Warren et al. [41] 2016 |  |  |  |  |  |  |  |  |  |  |

**Table S2.** **Newcastle-Ottawa scale scoring of studies included in the systematic review and meta-analysis.** * = Newcastle-Ottawa scale not applicable as randomised controlled trial.
